# A Long-Read Genome Assembly of a Native Mite in China *Pyemotes zhonghuajia* Yu, Zhang & He (Prostigmata: Pyemotidae) Reveals Gene Expansion in Toxin-Related Gene Families

**DOI:** 10.3390/toxins14080571

**Published:** 2022-08-21

**Authors:** Yan-Fei Song, Li-Chen Yu, Mao-Fa Yang, Shuai Ye, Bin Yan, Li-Tao Li, Chen Wu, Jian-Feng Liu

**Affiliations:** 1 State Key Laboratory Breeding Base of Green Pesticide and Agricultural Bioengineering, Key Laboratory of Green Pesticide and Agricultural Bioengineering, Ministry of Education, Guizhou University, Guiyang 550025, China; 2 Institute of Entomology, Guizhou University, Guizhou Provincial Key Laboratory for Agricultural Pest Management of the Mountainous Region, Scientific Observing and Experiment Station of Crop Pest Guiyang, Ministry of Agriculture, Guiyang 550025, China; 3 Changli Institute of Pomology, Hebei Academy of Agriculture and Forestry Sciences, Changli 066600, China; 4 College of Tobacco Science, Guizhou University, Guiyang 550025, China; 5 The New Zealand Institute for Plant and Food Research Limited, Auckland 1142, New Zealand

**Keywords:** *Pyemotes*, genome annotation, protein, toxin

## Abstract

*Pyemotes zhonghuajia* Yu, Zhang & He (Prostigmata: Pyemotidae), discovered in China, has been demonstrated as a high-efficient natural enemy in controlling many agricultural and forestry pests. This mite injects toxins into the host (eggs, larvae, pupae, and adults), resulting in its paralyzation and then gets nourishment for reproductive development. These toxins have been approved to be mammal-safe, which have the potential to be used as biocontrol pesticides. Toxin proteins have been identified from many insects, especially those from the orders Scorpions and Araneae, some of which are now widely used as efficient biocontrol pesticides. However, toxin proteins in mites are not yet understood. In this study, we assembled the genome of *P. zhonghuajia* using PacBio technology and then identified toxin-related genes that are likely to be responsible for the paralytic process of *P. zhonghuajia*. The genome assembly has a size of 71.943 Mb, including 20 contigs with a N50 length of 21.248 Mb and a BUSCO completeness ratio of 90.6% (n = 1367). These contigs were subsequently assigned to three chromosomes. There were 11,183 protein coding genes annotated, which were assessed with 91.2% BUSCO completeness (n = 1066). Neurotoxin and dermonecrotic toxin gene families were significantly expanded within the genus of *Pyemotes* and they also formed several gene clusters on the chromosomes. Most of the genes from these two families and all of the three agatoxin genes were shown with higher expression in the one-day-old mites compared to the seven-day-pregnant mites, supporting that the one-day-old mites cause paralyzation and even death of the host. The identification of these toxin proteins may provide insights into how to improve the parasitism efficiency of this mite, and the purification of these proteins may be used to develop new biological pesticides.

## 1. Introduction

*Pyemotes zhonghuajia* Yu, Zhang & He (Prostigmata: Pyemotidae) is a native viviparous mite initially collected from *Sinoxylon japonicum* Lesne and *Phloeosinus hopehi* Schedl by Lichen Yu in the 1990s [1]. It distributes naturally in Shanxi, Xinjiang, Ningxia, Hebei, Tianjin, and Beijing, China [2]. *Pyemotes zhonghuajia* is a dominant efficient ectoparasitic mite and is regarded as an important natural enemy in controlling many agricultural and forest pests [3]. This mite can inject toxins through puncturing the intersegmental cuticle using its mouthpart to paralyze a large number of pests including Isoptera, Homoptera, Hymenoptera, Lepidoptera, and Coleoptera [4,5,6,7]. After the host is completely paralyzed, the mite finds an optimal position to settle down and then obtains nourishment for reproductive development [7]. The gland of *P. zhonghuajia* is located at the junction of the head and neck, and those toxins are produced in secretory cells in the gland. The toxins produced by the mite are highly efficient in paralysis. A one-day-old mite *P. zhonghuajia* female can kill a third *Spodoptera litura* instar larva heavier than 680,000 times its own weight [7,8,9]. One single *P. zhonghuajia* female can lead to over 50% mortality rate of the first to third instar larvae of *Mythimna separata* Walker and *Spodoptera frugiperda* (Smith) [3,6]. The toxins are also found to be safe to mammals, which have the potential to be used as biological pesticides [6]. Nowadays, *Pyemotes zhonghuajia* is mass-produced and commercialized to control *M. separata*, *Aphis citricola* van der Goot, *S. frugiperda* Smith, *Sinoxylon japonicus* (Motschulsky)*, Monochamus alternatus* Hope, and *Zeuzera leuconotum* Bulter [3,4,6,10,11,12,13].

Toxin proteins from the order Scorpiones and Araneae have been widely studied, some of which are now used for pest control. The scorpion insect-specific neurotoxins *AaIT* and *LqhIT2* target the insect’s voltage-gated sodium channels that bind to the host’s motor nerve branches to play critical roles in electrical signaling in stimulating skeletal muscles [14]. Identified from the highly toxic scorpion *Androctonus australis* [15], *AaIT* is a single-chain polypeptide containing 70 amino acids and four disulfide bridges [16]. As it is safe to mammals, *AaIT* can be used for pest control [17,18]. *AaIT*-expressed baculovirus has shown to reduce the survival time of the pests coupled with a significantly enhanced infection efficiency of this virus [19]. For example, *AaIT*-expressed *Bombyx mori* nucleopolyhedrovirus (BmNPV) can reduce feeding damage from silkworms to the host, and introducing *AaIT* gene into entomopathogen *Beauveria bassiana* can enhance its virulence to mosquitoes [14]. *LqhIT2* is another protein known with the potential of pest control. It was isolated from the scorpion *Leiurus quinquestriatus hebraeus*, which comprises 61 amino acids with four disulphide bridges [17]. The feeding capacity of the rice leafroller (*Cnaphalocrocis medinalis* Guenee) was shown to be decreased in the *LqhIT2*-inserted transgenic rice compared to the wild type [17]. In Araneae, more than 800 toxins have been isolated and described [20]. Dermonecrotic toxins, well-characterized from the Brown spider (*Genus Loxosceles*), are biochemical constituents in spider crude venom, and it can also induce necrotic and dermonecrotic lesions on rabbits and mice [21]. Agatoxins are neurotoxins, identified from *Agelenopsis aperta* and classified into three classes (α-, μ-, and ω- agatoxins), specifically targeting three classes of ion channels (voltage-activated calcium channels, transmitter-activated cation channels, and voltage-activated sodium channels), respectively [22]. The α-agatoxins have an acylpolyamine structure and can induce immediate but reversible paralysis. The μ-agatoxins do not have this structure and cause immediate but irreversible paralysis. The ω- agatoxins divide into four types (ω-Aga-1A, ω-Aga-IIA, Type III ω-Agatoxins, and Type IV ω-Agatoxins), and inhibit voltage-activated calcium channels in nerve terminals. Some enzymes also play important roles in arachnid biotoxins. Identified from spider venoms, enzymes mainly serve two important functions: (1) lysing polymers in the extracellular matrix and (2) binding to the compounds in the membrane [23,24].

There are only a few studies on toxin proteins in mites. In straw itch mite (*Pyemotes tritici*), a low molecular weight protein TxP was identified and showed inducing a rapid, muscle-contracting paralysis [25]. The toxicity of TxP -1 has shown to be comparable to *AaIT* or even stronger [26]. The TxP proteins were found to be translated by a range of cDNAs with variable length that are homologs to the insect-selective paralytic neurotoxin *tox34* [25]. It is notable that more than 18 recombinant baculoviruses engineered with *tox34* have been used for pest control [19,26]. In *P. zhonghuajia,* 12 *tox34* homologs were identified with sequence similarity ranging from 84.21% to 90.42% compared with those found in *P. tritici* [9]. However, other than these sequences, there is little genetic information about toxin-related proteins in the genus of *Pyemotes* that contains many venomous ectoparasitic mites.

Here, we sequenced the genome of *P. zhonghuajia* using Pacific Bioscience technology on the single-molecule real-time (SMRT) platform. The assembled genome was annotated with protein-coding genes, repeats, and non-coding RNAs (ncRNAs). We analyzed gene family evolution across Arachnida (1 Araneae, 1 Scorpiones, and 10 Acarina) with the main focus on the toxin-related genes.

## 2. Results and Discussion

### 2.1. Genome Assembly

A total of ~13 Gb and ~11 Gb Illumina short reads (150 bp) and PacBio long reads (average length of 9699.12 bp and N50 of 12.537 kb) were generated for genome assembly. The k-mer analysis based on short reads estimated genome size of 69.33 Mb comprising 7.09 Mb repetitive regions with genome heterozygosity of 0.04% (Supplementary Table S1). The *P. zhonghuajia* genome was assembled into 19 scaffolds containing 71.943 Mb with N50 of 21.248 Mb (Table 1), and 68.511 Mb was assigned to three pseudo-chromosomes. This assembled genome has a size comparable to the k-mer estimation and achieved a BUSCO complete gene ratio of 90.6% with duplicated and missing gene ratios of 1.3% and 7.9%, respectively. The mapping back rates from short and long reads as well as RNA-Seq data were 98.44%, 96.72%, and 92.84%, respectively. Compared with the other two mite genome assemblies containing thousands of scaffolds/contigs, our *P. zhonghuajia* genome assembly is much more continuous (Table 1). This genome assembly has a comparable size with the other two mite genomes.

### 2.2. Genome Annotation

There was 11.4% of the assembly annotated as repetitive regions, which contained 77.050 repeats taking up ~8.2 Mb. The most abundant repeat class was LTR elements, taking up 3.62% of the assembly, followed by simple repeats (2.92%), unclassified repeats (1.98%), low complexity repeats (1.42%), and DNA elements (0.79%) (Supplementary Table S2). There were 11,183 gene models annotated with the average length of 3243.42 bp and 3.59 exons per gene. The average lengths of exon and intron were 475.53 bp and 626.12 bp, respectively. The BUSCO result showed 91.2% complete genes with duplicated and missing genes being 2.5% and 6.5%, showing that most of the annotated genes are likely to have complete lengths. It is noticed that the BUSCO complete gene ratio resulted from the annotated gene set is slightly higher than the genome assembly. It is likely because BUSCO only employs AUGUSTUS for gene prediction and has less power to predict complete genes correctly compared with our gene annotation method that was supported by RNA-Seq data. [27]. There were 218 ncRNAs annotated in the genome assembly, including eight miRNA, 48 rRNA, nine snRNA, and 112 tRNA. The annotated snRNAs included five spliceosomal RNAs (U1, U2, U4, U5 and U6), one minor splicesomal RNA (U6atac), and three C/D box snoRNAs (U3 and snoR38) (Supplementary Table S3). The tRNAs Supres and SelCys were absent in the annotation. This is the first non-coding RNA set annotated in a mite genome.

### 2.3. Species Phylogeny and Gene Family Evolution

**Figure 1:**
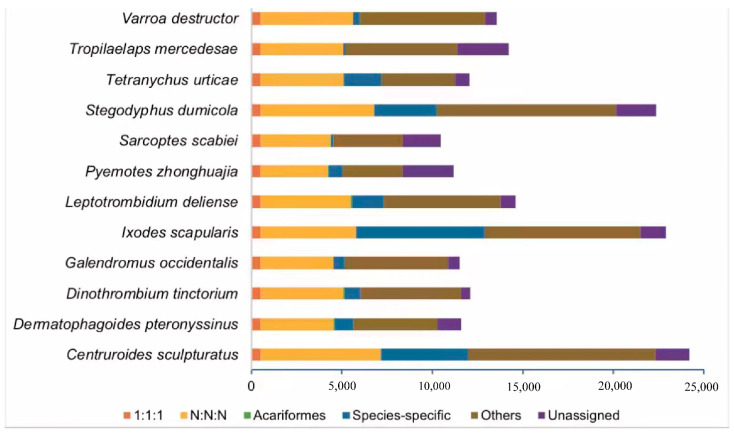
Histogram shows the number of genes assigned to different groups. The “1:1:1” and “N:N:N” groups represent single- and multi-copy genes found in all the species. The group “Acariformes” represents orthologs unique to Acariformes. The “Others” group indicates other orthologs which do not belong to any above-mentioned ortholog categories. The group “Unassigned” represent the orthologs which can’t be assigned to any orthogroups.

There were 12 species including *P. zhonghuajia* selected for phylogenetic construction. In total, 180,720 genes were clustered into 17,407 gene families. In *P. zhonghuajia,* a total of 11,183 genes were analyzed, and there were 8388 genes assigned into 6224 gene families with species-specific gene families and genes being 178 and 761, respectively (Figure 1).

**Figure 2:**
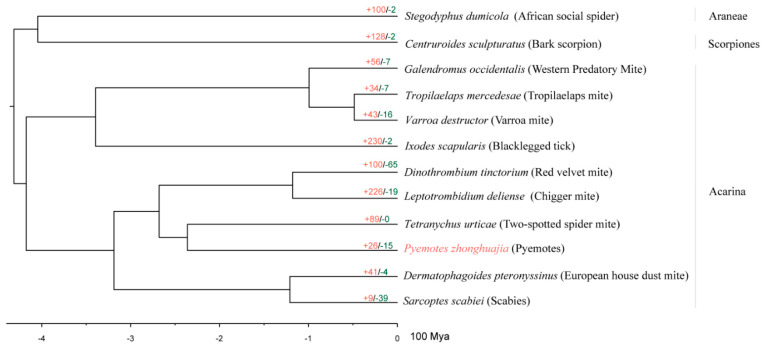
Dating tree with node values representing the number of expanded, contracted.

There were 527 single-copy genes found in all the species, 473 of which contained 143,759 amino acid sites were used to construct a phylogenetic tree. All node supports were 100/100 (SH-aLRT support /ultrafast bootstrap support). The phylogenetic tree shows that *P. zhonghuajia* is sister to the two-spotted spider mite (*T. urticae*) and they are clustered with the other two mite species: chigger mite (*Leptotrombidium delicense*) and red velvet mite (*Dinothrombium tinctorium*) (Figure 2). The phylogenetic tree was consistent with the published classifications [1] and our calculation indicated *P. zhonghuajia* together with *T. urticae* emerged during Triassic (223.43~247.66 Mya).

**Figure 3:**
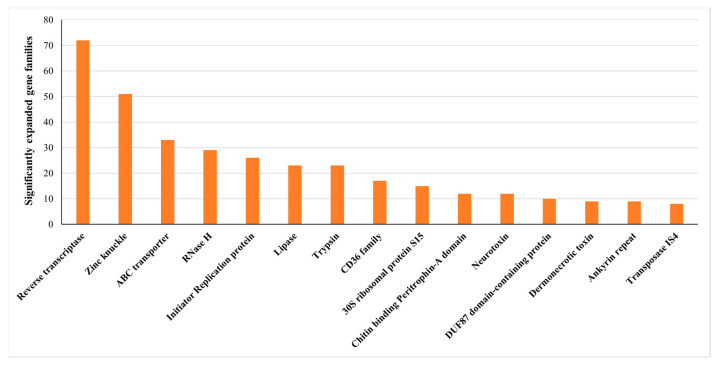
Top fifteen significantly expanded families with gene numbers of the families shown above the bars.

Compared with the closely related *T. urticae* that has over one thousand gene families identified as expanded families, *P. zhonghuajia* only has 621(containing 1224 genes) gene families that were calculated as expanded families (Figure 2 and Figure 3). These families are related to digestion, detoxification, and toxins, and those such as ABC transporter, Lipase, Trypsin, Dermonecrotic toxin, CD36 family, and neurotoxins (Supplementary Table S4). The families of dermonecrotic toxins and neurotoxins are mostly likely to participate in the parasitic mechanism [25,28,29,30,31]. There were 3927 genes lost in 3664 contracted families, including 15 gene families that were identified as significantly contracted families. These contracted families might be associated with viviparous reproduction in pyemotid mite; no free-living stages of larvae and nymph occur during the life cycle of *P. zhonghuajia* (Supplementary Table S5) [32].

The results from the enrichment analysis of GO terms and KEGG pathways also showed the expanded gene families belong to the categories of digestion, such as lipid metabolic process and phosphatidic acid biosynthetic process (Supplementary Figure S1A), and PPAR signaling pathway and Biosynthesis of unsaturated fatty acids (Supplementary Figure S1B). This suggests some of the gene family expansions are likely to be involved in the extensive feeding habits. The expanded chitin-binding families (Supplementary Figure S1A) may be related to intense enlargement of the parasitoid body during the reproductive period [33].

### 2.4. Neurotoxin, Dermonecrotic Toxin and Agatoxin Genes

**Figure 4:**
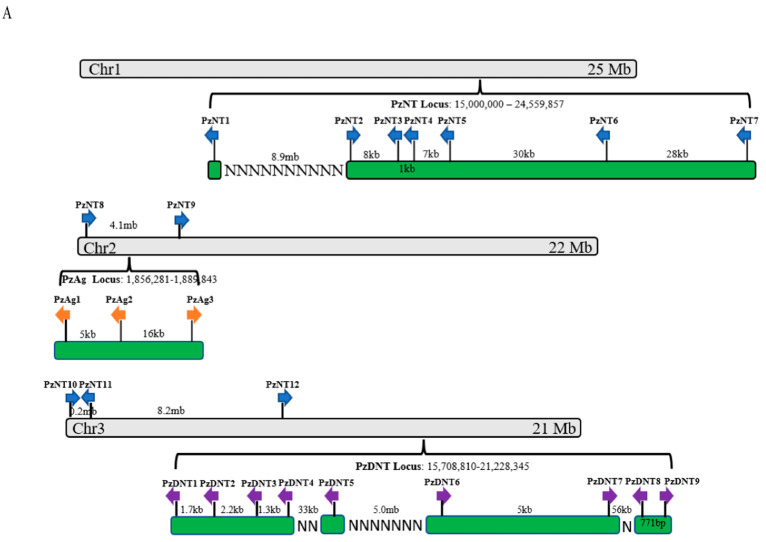

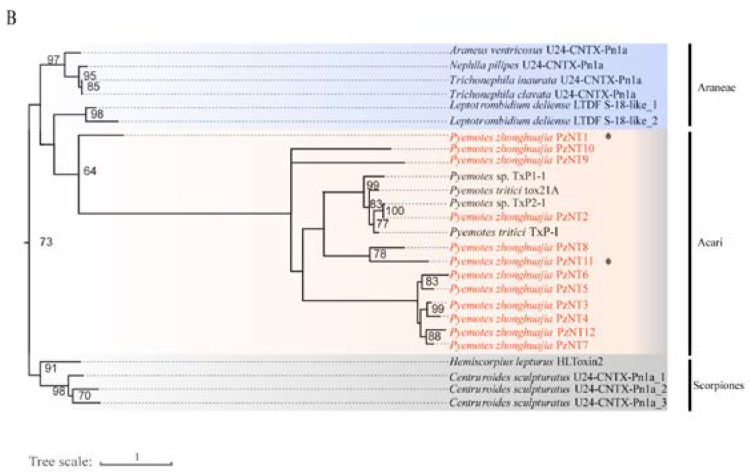

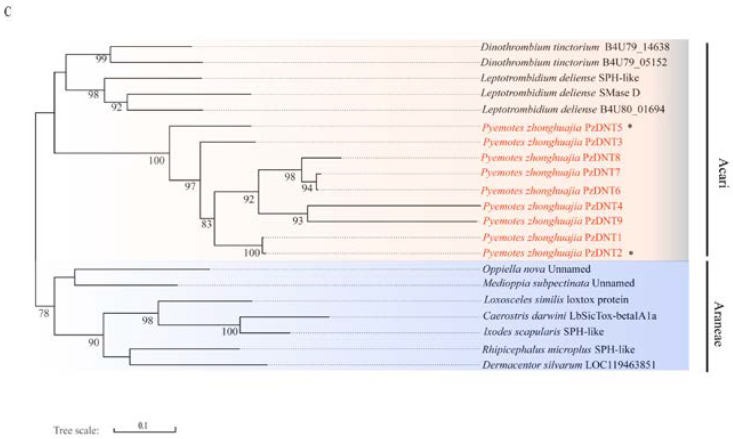

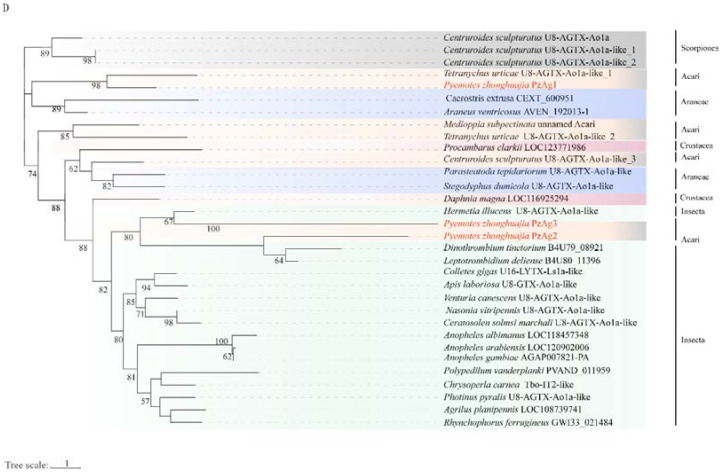
Distribution of toxin genes on the chromosomes and phylogenetic trees of the three toxin gene classes. (**A**) Distribution of toxin genes on the chromosomes. *Blue*: neurotoxin genes; *Orange*: agatoxin genes; *Purple*: dermonecrotic toxin genes. (**B**–**D**) Phylogenetic trees of neurotoxin genes, dermonecrotic toxin genes, and agatoxin genes. The “*” indicates the gene was shown with higher expression in the seven-day-pregnant mites compared with the one-day-old mites. The accession numbers of all the sequences used in the phylogenies are listed in Supplementary Table S12.

It is interesting that we found significant expansion of neurotoxin and dermonecrotic toxin gene families in the *P. zhonghuajia* genome. Neurotoxins in spiders were found as the main component of the venom that targets the prey’s ion channels leading to paralysis or death [34]. The *P. zhonghuajia* neurotoxin gene family was expanded within the Acari clade (Figure 4B). The 12 neurotoxin genes were present on all the three chromosomes with a six-gene locus being located near the end of chromosome 1 (Figure 4A). *PzNT3* and *4* were located nearby and closely related on the phylogenetic tree with nearly 85% sequence similarity (Supplementary Table S6), suggesting they were recently duplicated under a tandem duplication event. Similar observations were from *PzNT5* and *6*. Located on the two different chromosomes, a recent duplication event might have also occurred between *PzNT7* and *12* as they were closely related on the phylogenetic tree with high sequence similarity. *PzNT2* was present as a homologous sequence to *P. tritici* TxP2-1 (80% sequence similarity) and they were closely related to *P. tritici* TxP-1 (endcoded by *Tox34*) that was found playing a role in paralyzing and even killing insects [26,35]. Most neurotoxin genes (except *PzNT1* and *11*) have a higher number of reads aligned from RNA-Seq data obtained from the one-day-old mites compared with the seven-day-pregnant mites, indicating higher levels of gene expression (Supplementary Figure S2). *PzNT1* was located distantly from the six-gene locus on chromosome 1 and had shown to be distantly related to the rest of the neurotoxin genes on the phylogenetic tree. It is possible that there was a functional divergence of this gene compared with the rest of the family members.

Dermonecrotic toxins have been demonstrated to cause dermonecrotic lesions on the prey in spiders [21] and it is likely that the dermonecrotic toxins of *P. zhonghuajia* may also cause the dermonecrotic lesion of the host. In *P. zhonghuajia*, all nine dermonecrotic toxin genes were present roughly close to each other on the second half of chromosome 3 (Figure 4A). Our phylogenic tree showed these genes formed a single clade with the gene family expansion occurring after Pyemotes separated from Leptotrombidium and Dinothrombium (Figure 4C). *PzDNT1* and *2* were located next to each other and showed to be closely related on the phylogenetic tree with over 97% sequence similarity (Supplementary Table S8), suggesting a recent tandem gene duplication event. However, their expression divergence (higher expression of *PzDNT1* in the one-day-old mites compared with seven-day-pregnant mites and *PzDNT2* is opposite) suggests a functional difference. A recent duplication event was also shown between *PzDNT6* and *7*, both of which exhibited similar expression patterns in the two adult forms. Similar to *PzDNT2*, *PzDNT5* was also shown with higher expression level in the seven-day-pregnant mites compared with the one-day-old mites.

α-agatoxins was found to paralyze the prey in funnel web spiders [22]. There were three agatoxin genes identified in the *P. zhonghuajia* that formed a gene cluster on chromosome 2, which, with each other, has shown relatively distantly related on the phylogenetic tree (Figure 4D). *PzAg2* and *3* were separated from *PzAg1* at a very early stage during evolution. All three genes exhibited higher expression in the one-day-old mites compared with seven-day-pregnant mites, suggesting a role in paralyzing the hosts.

## 3. Conclusions

Pyemotidae is a significant family with several species being natural enemies, such as *P. tritici* and *P. zhonghuajia.* They can paralyze and even cause the death of the stored product insects and agriculture and forestry insects. Understanding the composition and function of toxin-related proteins of *P. zhonghuajia* is crucial to improve its predation efficiency and it can also provide the knowledge for the potential of transferring the mite toxin-related genes into crop genomes for pest control. In this study, we have generated a chromosome-level genome assembly of *P. zhonghuajia*, which is the first whole-genome assembly in Pyemotidae that provides an important genomic resource for the study of biocontrol potential and ecological importance. The two gene families encoding neurotoxins and dermonecrotic toxins were found with significant expansion within the genus of *Pyemotes*, which also formed several gene clusters on the chromosomes. It is possible that gene expansion provides a high dosage of toxin proteins that are released during parasitic process. Gene expansion might also result in a highly diverged population of toxins that enable the mite to have a wide range of hosts. Several recent gene duplication events that we observed from the two gene phylogenies indicate they may underlie key adaptive events in the evolution of *P. zhonghuajia*. All the toxin-related genes including the three agatoxin genes were shown with expression in the adults, and most of them exhibited higher level of expression in the one-day-old mites compared with seven-day-pregnant mites. This matches our observations on the one-day-old mite forms paralyzing and killing the hosts such as *S. frugiperda*, *M. separata*, and *S. litura* [3,6,7]. Future research will focus on confirming the presence of the proteins encoded by these genes through proteomic studies and functional characterization of the proteins through protein purification approaches and feeding experiments in *P. zhonghuajia*. Similar studies will also be conducted in other venomous ectoparasitic species from *Pyemotes*.

## 4. Materials and Methods

### 4.1. Sample Collection and Sequencing

Colonies of *P. zhonghuajia* were reared on mature larvae of *Sitotroga cerealella* (Oliver) (Lepidoptera: Gelechiidae) with wheat bran in a climate chamber at 25 ± 1 °C with 60 ± 5% relative humidity (RH) at Changli Institute of Pomology, Hebei Academy of Agriculture and Forestry Sciences. There were 2000 seven-day-pregnant mites used for Illumina whole-genome and PacBio sequencing, respectively. Genomic DNA was extracted using the QIAGEN DNeasy Blood & Tissue kit, which was then used to construct a 350 bp insert-size library using the Truseq DNA PCR-free kit for sequencing on the Illumina NovaSeq 6000 platform and a 15 kb insert-size library using the SMRTbell™ Template Prep Kit 2.0 for sequencing on the PacBio Sequel II platform. The whole-individual transcriptome was performed using RNA-Seq from 2000 one-day-old mites and 2000 seven-day-pregnant mites with three biological replicates for each group. Total RNA was extracted using the TRIzol™ Reagent kit and the RNA-Seq library was constructed using TruSeq RNA v2 kit. DNA/RNA extraction, library construction, and sequencing were performed at Berry Genomic (Beijing, China).

### 4.2. Genome Assembly

Illumina raw reads were cleaned using two tools under BBTools v38.67 [36] with the following steps: (1) removing duplicated reads; (2) trimming low-quality reads; (3) removing poly-A/G/C tails; (4) filtering reads less than 15 bp; and (5) correcting reads based on overlapping ends between pairs. The tool Clumpify was used for step (1) and steps (2) to (5) were performed using BBDuk with parameters “qtrim = rl trimq = 20 minlen = 15 ecco = t maxns = 5 trimpolya = 10 trimpolyg = 10 trimpolyc = 10”. K-mer analysis based on Illumina short reads was performed using BBNorm (*k-mer*: 21) and the k-mer profile was visualized using the online version of Genomescope v2.0 [37] with parameters “-k 21 -p 2m 1000”. A preliminary PacBio long-read assembly was performed using Flye v2.7.1 [38] with parameters “-i 2-m 3000”. Purge_Dups v1.0.0 [39] was used to remove allelic contigs based on the read depth with a minimum alignment score of 70 after the long reads were mapped back to the assembly with Minimap2 (v2.17) [40]. Illumina short reads were used for two rounds of contig polishing performed by NextPolish (v1.1.0) [41] after mapping the reads back to the assembly using Minimap2. The contaminated contigs were assessed and removed using “blastn” from BLAST+ (v2.9.1) [42] with the sequence similarity search against *nt* and UniVec databases (both were downloaded in December 2020). The cleaned contigs were then uploaded to NCBI for an additional check of contamination. Assembly completeness was estimated using BUSCO (v3.1.0) [43] with the sequence similarity search against the arthropod single-copy gene set (arthropoda_odb9: n = 1367). To estimate the mapping rate from raw reads, both short and long genomic reads as well as RNA-Seq short reads were aligned back to the genome assembly using Minimap2.

### 4.3. Genome Annotation

Three essential genomic elements of *P. zhonghuajia* genome: repetitive elements, non-coding RNAs (ncRNAs), and protein-coding genes were annotated. To annotate repeats, RepeatModeler v2.0.1 [44] with LTR search process (-LTRStruct) was used to generate a de novo repeat library, which was then combined with Dfam 3.1 [45] and RepBase-20181026 [46] databases to form a custom repeat library. The repeat-masked genome assembly was produced using RepeatMasker v4.0.9 [47].

The MAKER v2.31.10 [48] pipeline was used to predict protein-coding genes by integrating ab initio, transcript- and homology-based evidence. The ab initio prediction was generated by a BRAKER v2.1.5 [49] pipeline to train Augustus v3.3.3 [50] and GeneMark-ES/ET/EP 4.48_3.60_lic [51] with the utilization of RNA-Seq data and protein sequences to increase prediction accuracy. The input alignments from mapping RNA-seq data to the genome assembly were produced using HISAT2 v2.2.0 [52] and the arthropod protein sequences were obtained from OrthoDB10 v1 database [53]. The genome-guided assembler StringTie v2.1.2 [54] was used to assemble transcripts as transcriptome evidence to integrate in MAKER. The protein sequences that were also utilized by MAKER for final prediction were downloaded from NCBI including the sequences from *Drosophila melanogaster* (Insecta), *Daphnia magna* (Crustacea), *Ixodes scapularis*, *Varroa destructor*, *Tetranychus urticae*, and *Dermatophagoides pteronyssinus* (Acari). Gene functional annotation was performed using Diamond v0.9.24 [55] from searching against the UniProtKB database with the sensitive mode “--more-sensitive -e 1e^−5^”. InterProScan 5.41–78.0 [56] was used to search against the databases including Pfam [57], SMART [58], Gene3D [59], Superfamily [60] and CDD [61]. The protein domains, gene ontology (GO), and gene pathways (KEGG, Reactome) were annotated using ggnog-mapper v2.0.1 [62] from searching against ggnog v5.0 [63].

NcRNAs including rRNA, snRNA, and miRNA were identified using infernal v1.1.3 [64] by searching sequence similarity against Rfam database. TRNAs were predicted using tRNAscan-SE v2.0.6 [65] and the high-confident sequences were maintained as the final tRNA set by the tRNAscan-SE script “EukHighConfidenceFilter”.

### 4.4. Gene Ontology Analysis and Species Evolution

The protein sequences from 11 species across three orders (Araneae: Stegodyphus dumicola; Scorpiones: Centruroides sculpturatus; Arachnoidea: Dermatophagoides pteronyssinus, Dinothrombium tinctorium, Galendromus occidentalis, Ixodes scapularis, Leptotrombidium deliense, Sarcoptes scabiei, T. urticae, Tropilaelaps mercedesae, and Varroa destructor) were downloaded from NCBI in December 2020 together with the annotated protein set from *P. zhonghuajia* for gene family orthology inference using OrthoFinder v2.3.8 [66] after aligning sequences using Diamond. The resulting single-copy orthologs from OrthoFinder were used for phylogenetic analysis. The sequence alignment as input for phylogenetic construction was generated using the following steps: (1) aligning orthologous protein sequences using MAFFT v7.394 [67] with “L-INS-I”; (2) filtering ambiguous aligned regions using BMGE v1.12 [68] with the parameters “-m BLOSUM90 -h 0.4”; (3) Concatenating all the protein alignments generated above using FASconCAT-G v1.04 [69] as the input for phylogenetic construction. The species phylogeny was constructed using IQ-TREE v2.0-rc2 [70] with the parameters “-m MFP --mset LG --msub nuclear–rclusterf 10 -B 1000 --alrt 1000 --symtest-remove-bad --symtestpval 0.10”. The estimated time of species divergence was calculated using MCMCTree from PAML v4.9j [71] with parameters “clock = 2, BDparas = 1 1 0.1, kappa_gamma = 6 2, alpha_gamma = 1 1, rgene_gamma = 2 20 1, sigma2_gamma = 1 10 1”. There were four fossil evidences downloaded from the PBDB database (https://www.paleobiodb.org/navigator/, accessed on 14 August 2022) as calibrations used in this estimation above: Allopalaeophonus caledonicus (4.305–4.438) from the order Scorpiones as the root, Pseudoprotacarus scoticus (4.076–4.192) from Arachnida, Carbolohmannia maimaiphilus (3.114–3.232) from Acariformes, and Deinocroton draculi (0.935–1.455) from Mesostigmata.

### 4.5. Identification of Gene Family Expansion and Contraction

The gene family expansion and contraction in *P. zhonghuajia* genome compared with the 11 species used for phylogenetic construction were estimated using CAFÉ v4.2.1 [72] with the model of single birth–death parameter lambda and a significance level of 0.01 (*p* = 0.01). The identified significantly expanded gene families were then assigned with GO and KEGG categories using R package clusterProfiler v3.10.1 [73] with the default parameters (*p* = 0.01 and *q* = 0.05).

### 4.6. Phylogeny Construction and Gene Expression of Toxin-Related Gene Families

The *P. zhonghuajia* neurotoxin, dermonecrotic toxin, and agatoxin protein sequences were predicted from the genome assembly using BITACORA v1.2 [74] based on homology searches using the sequences from Chelicerata and Myriapoda downloaded from NCBI RefSeq (Supplementary Table S11) and confirmed by searching against protein database using the online blastp (https://blast.ncbi.nlm.nih.gov/Blast.cgi?PROGRAM=blastp&PAGE_TYPE=BlastSearch&LINK_LOC=blasthome, accessed on 14 August 2022). The HMM profiles generated from HMMER v3.2.1 [75] using “hmmbuild” were used in BITACORA. Multiple alignments were performed using Geneious prime V 2021.1.1 (created by Biomatters. Available from https://www.geneious.com, accessed on 5 November 2020) with the method of Clustal Omega. FastTree [76] was used to construct neurotoxin, dermonecrotic toxin, and agatoxin protein phylogenies based on the maximum likelihood method. We used RNA sequencing data to detect the expression of toxin-related genes in seven-day-pregnant mites and one-day- old mites. The estimation of gene expression levels was performed using Salmon [77] with the Gaussian axial fluctuation (GAF) model to generate normalized read counts. The gene expression heatmap was generated using the heatmap function (heatmap) from the R package NMF [78] (Supplementary Figure S2).

## Figures and Tables

**Table 1:** Genome Assembly and Annotation Statistics of *P. zhonghuajia*, *Tetranychus urticae*, and *Dermatophagoides pteronyssinus*.

Elements	*Pyemotes zhonghuajia*	*Stratiolaelaps scimitus*	*Tetranychus urticae*
Genome assembly			
Assembly size (Mb)	71.943	426.50	89.6
Number of scaffolds/contigs	19/20	158	-/2035
Longest scaffold/contig (Mb)	25.136/22.128	31.29	7/0.929
N50 scaffold/contig length (Mb)	22.128/21.248	7.66	10/120
GC (%)	25.02	45.85	-
Gaps (%)	0.00	0.00	-
BUSCO completeness (%)	90.6	93.1	-
Annotation			
Protein-coding genes	11,183	13,305	18,414
Mean protein length (aa)	480.97	500.59	-
Mean gene length (bp)	3243.42	7870.13	2652
Exons/introns per gene	3.59/2.45	6.24/-	3.82/-
Exon (%)	26.77	7.25	-
Mean exon length	475.53	372.35	178
Intron (%)	24.07	5.02	-
Mean intron length	626.12	1105.66	400
BUSCO completeness (%)	91.2	95.8	-

## Data Availability

Genome assembly and raw sequencing data have been deposited at the NCBI under the accessions JACCHO000000000 and SRR12261228—SRR12261230, respectively. Genome annotations are available at the Figshare under the link: https://figshare.com/articles/dataset/A_Long-Read_Genome_Assembly_of_a_Native_Mite_in_China_Pyemotes_Zhonghuajia_Yu_Zhang_He_Prostigmata_Pyemotidae_Item/20518008 (accessed on 18 August 2022).

## Supplementary Materials

The following are available online at https://www.mdpi.com/article/10.3390/toxins14080571/s1, Table S1: Genome survey based on k-mer distribution for *pyemotes zhonghuajia* (excel file), Table S2: Repeat annotation in the *Pyemotes zhonghuajia* genome (excel file), Table S3: Annotations of non-coding RNAs in the *Pyemotes zhonghuajia* genome (excel file), Table S4: Significantly expanded gene families (excel file), Table S5: Expended and contracted gene families in each species. Table S6: The identity of neurotoxins of *P. zhonghuajia* with *P. tritici* (excel file), Table S7: The identity of agatoxins of *P. zhonghuajia* with other species (excel file), Table S8: The identity of dermonecrotic of *P. zhonghuajia* with other species (excel file), Table S9: Information of the 24 toxin-related genes, Table S10:Transcript per million (TPM) values (excel file), Table S11: Gene IDs of the reference species, Table S12: The accession numbers of all the sequences used in the phylogenies (excel file). Figure S1: GO (A) and KEGG (B) function enrichment of significantly expanded gene families. Figure S2: Heatmap of the identified toxin-related genes (pdf file).
