# Supplementary material for: A Long-Read Genome Assembly of a Native Mite in China Pyemotes zhonghuajia Yu, Zhang & He (Prostigmata: Pyemotidae) Reveals Gene Expansion in Toxin-Related Gene Families
Source: Toxins (Basel). 2022 Aug 21;14(8):571. doi: 10.3390/toxins14080571 (PMC9415403; doi:10.3390/toxins14080571)
Supplement: Supplementary file 1 [file toxins-14-00571-s001.zip › Figure S1.pdf]

(a)

(b)

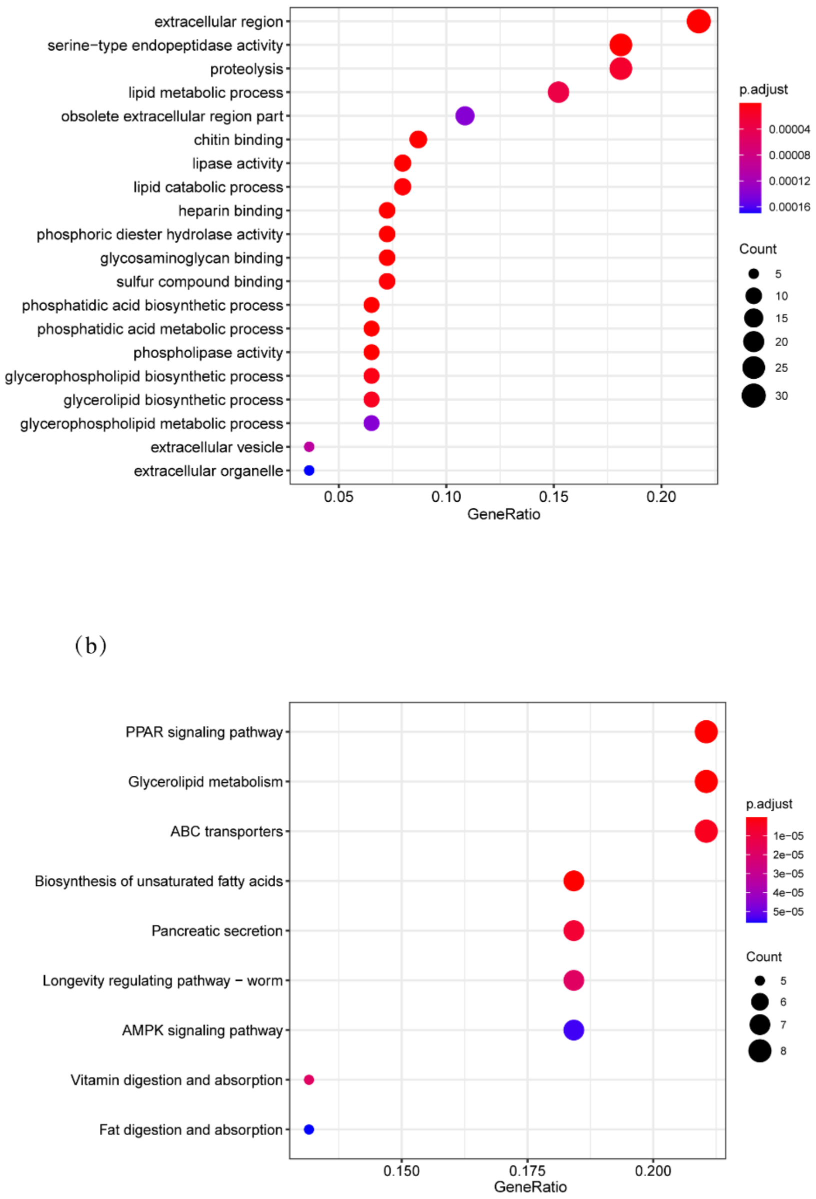

Figure S1: GO (A) and KEGG (B) function enrichment of significantly expanded gene families. Only the top 20 categories are shown.
