# Supplementary figures and images for: A Long-Read Genome Assembly of a Native Mite in China Pyemotes zhonghuajia Yu, Zhang & He (Prostigmata: Pyemotidae) Reveals Gene Expansion in Toxin-Related Gene Families
Source: Toxins (Basel). 2022 Aug 21;14(8):571. doi: 10.3390/toxins14080571 (PMC9415403; doi:10.3390/toxins14080571)

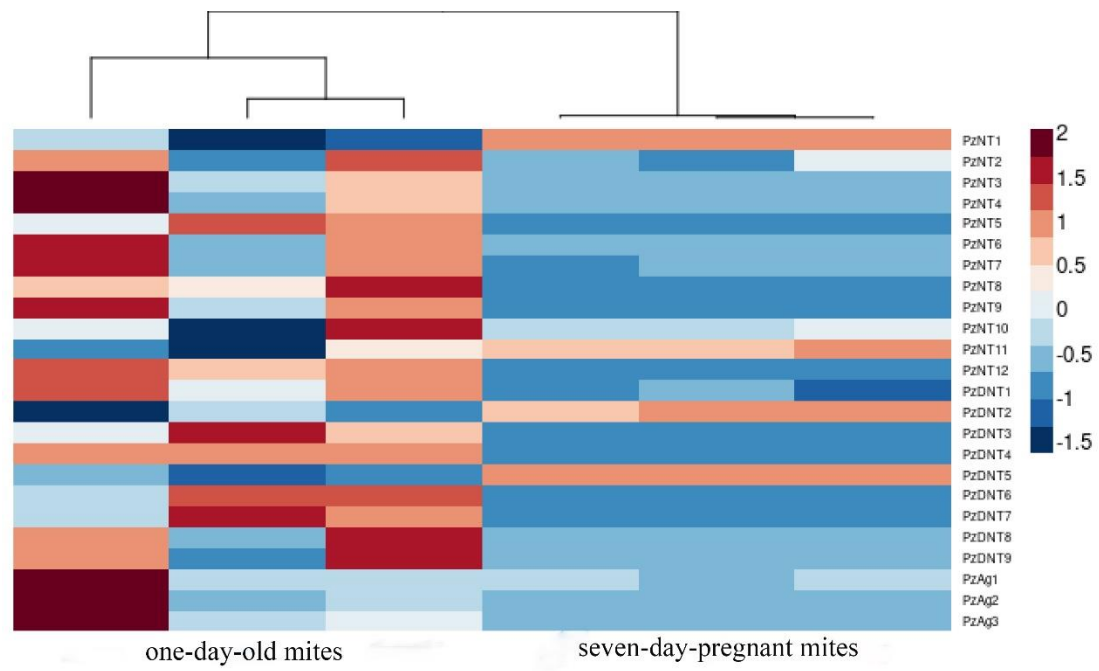

Figure S2: Heatmap of the identified toxin-related genes.

Supplement: Supplementary file 1 [file toxins-14-00571-s001.zip › Figure S2.pdf]
